# Apparatus for attosecond transient-absorption spectroscopy in the water-window soft-X-ray region

**DOI:** 10.1038/s41598-023-29089-8

**Published:** 2023-02-21

**Authors:** Kristina S. Zinchenko, Fernando Ardana-Lamas, Valentina Utrio Lanfaloni, Tran Trung Luu, Yoann Pertot, Martin Huppert, Hans Jakob Wörner

**Affiliations:** 1 Laboratory of Physical Chemistry, ETH Zürich, 8093 Zurich, Switzerland; 2 European XFEL GmbH, 22869 Schenefeld, Germany; 3 Department of Physics, The University of Hong Kong, Pokfulam Road, SAR Hong Kong, People’s Republic of China; 4 Paul Scherrer Institut, PSI, 5232 Villigen, Switzerland

**Keywords:** Attosecond science, Ultrafast photonics

## Abstract

We present an apparatus for attosecond transient-absorption spectroscopy (ATAS) featuring soft-X-ray (SXR) supercontinua that extend beyond 450 eV. This instrument combines an attosecond table-top high-harmonic light source with mid-infrared (mid-IR) pulses, both driven by 1.7–1.9 mJ, sub-11 fs pulses centered at 1.76 $\upmu$m. A remarkably low timing jitter of $\sim$ 20 as is achieved through active stabilization of the pump and probe arms of the instrument. A temporal resolution of better than 400 as is demonstrated through ATAS measurements at the argon L$_{2,3}$-edges. A spectral resolving power of 1490 is demonstrated through simultaneous absorption measurements at the sulfur L$_{2,3}$- and carbon K-edges of OCS. Coupled with its high SXR photon flux, this instrument paves the way to attosecond time-resolved spectroscopy of organic molecules in the gas phase or in aqueous solutions, as well as thin films of advanced materials. Such measurements will advance the studies of complex systems to the electronic time scale.

## Introduction

Since the discovery of the high-order harmonic generation (HHG) process^1,2^, countless efforts have been made to extend the cutoff energy of the harmonics, to generate the shortest isolated attosecond pulses, and to increase the photon flux^2–10^ of such light sources. These efforts are motivated by the broad range of applications that benefit from the unique characteristics of HHG sources, especially the ultrabroad spectral coverage and attosecond temporal resolution. These distinctive characteristics, combined with X-ray absorption spectroscopy, open the possibility of retrieving ultrafast dynamics with an unprecedented temporal resolution, element specificity and site selectivity.

High-harmonic generation has already proven to be a unique tool in different fields of ultrafast science, allowing to perform a broad range of table-top experiments, including element-specific dynamics with femtosecond time resolution in the gas^7^ and liquid phases^11^, photoionization delays in atoms^12,13^, molecules^14^, liquids^15^ and clusters^16^, or magnetization dynamics^17^.

In the present work, we provide a detailed description of the instrument that has been developed to observe fundamental processes such as charge transport or charge migration in molecules and complex systems with attosecond temporal resolution in the water-window energy regime. Section “Laser system” provides a brief description of the laser system used to drive the HHG process. Section “Soft-X-ray transient-absorption beamline” details the optical and vacuum setups for the HHG as well for guiding the SXR beam (probe) and the short mid-IR beam (pump) towards the sample under investigation. Section “High-resolution soft-X-ray spectrometer” describes the interaction chamber as well as the SXR spectrometer used to record the transient-absorption spectra. To conclude, section “Spectral and temporal resolution of the instrument” presents several measurements revealing the capabilities of our instrument. We present static absorption measurements on carbonyl sulfide (OCS), demonstrating the possibility of simultaneously detecting dynamics at multiple edges, specifically sulfur L$_{2,3}$- and the carbon K-edges. Additionally, we demonstrate the measurements of light-induced states in argon, which demonstrate the sub-femtosecond temporal resolution of the instrument.

## Laser system

### Description

**Figure 1:**
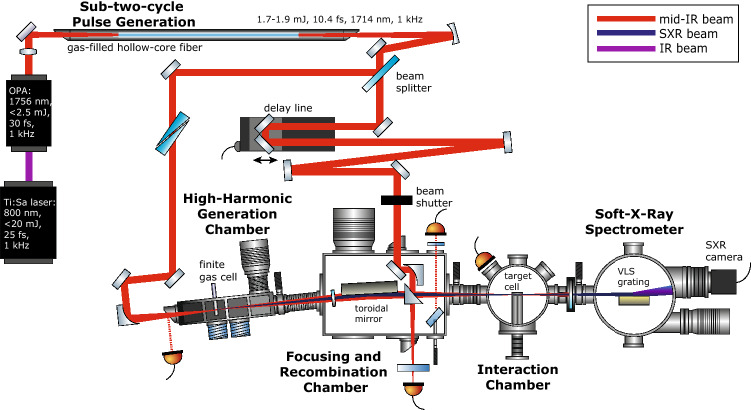
Experimental setup. Schematic diagram of the experimental setup for attosecond transient-absorption spectroscopy: finite gas cell (bottom left) for high-harmonic generation, pump-probe delay generation (middle), recombination, and attached interaction region (bottom middle), SXR spectrometer (bottom right). The spectrometer can be directly attached behind the recombination chamber for characterization of the generated SXR, or after the interaction region for transient-absorption measurements.

Ultrafast pump-probe measurements in the water-window region require a coherent light source with a long driving wavelength and high pulse energy. The schematic diagram of the femtosecond laser system and the experimental setup are shown in Fig. 1. The laser system consists of a regenerative and a cryogenically-cooled amplifier delivering 19 mJ, 28 fs pulses, centered at 800 nm at 1 kHz repetition rate. These pulses are used to pump a white-light-seeded optical parametric amplifier (OPA), generating mid-IR idler pulses with an average pulse energy of 2.5 mJ centered at 1.76 $\upmu$m.

### Production of sub-two-cycle mid-infrared pulses

Chirped-pulse amplification systems lack the capability to directly provide few-cycle pulses because of gain narrowing in the amplification step. Therefore, common schemes to generate intense (multi-mJ) few-cycle pulses rely on external compression in a hollow-core fiber (HCF)^18–21^ or filamentation^22–24^ subsequent to the laser system. The hollow-core fibers, filled with noble gases, have become an established pulse-compression technique paving the way to new applications in the field of extreme nonlinear light-matter interaction where the electric field of the laser pulse, rather than its intensity profile, is relevant. Thus, in our case, tailoring the electric field on electronic timescales, which is essential for the generation of single attosecond pulses and measurements of attosecond dynamics, requires the coherent superposition of electromagnetic waveforms with more than one octave of spectral bandwidth. Therefore, the mid-IR idler pulses from the OPA are first spectrally broadened in an argon-filled hollow-core fiber and then consecutively compressed with bulk material. Pulse durations of less than two optical cycles (10.4 fs) with 76$\%$ energy throughput have been obtained in this way^24^. These pulses are passively carrier-envelope-phase (CEP) stable due to the white-light-seeded OPA used in this work^25^.

## Soft-X-ray transient-absorption beamline

After the hollow-core fiber, the mid-IR beam is coupled into a Mach–Zehnder interferometer, as is shown in Fig. 1 including its optical and vacuum systems. First, it is recollimated with a concave silver mirror and then split into the pump and probe beams by a partially reflective mirror (R70:T30), hereinafter referred to as a beam splitter (BS). It covers the wavelength range between 1 and 2.2 $\upmu$m, sufficient for the entire bandwidth of our mid-IR pulse. The pump beam, transmitted through the BS, is used to excite the sample, whereas the probe beam, reflected from the BS, is used to generate the SXR continuum to probe the dynamics induced by the pump pulses. Ultraviolet-grade fused-silica wedges are inserted into the beam paths to finely compensate for the positive dispersion of the pump and probe pulses independently.

The pump and probe arms of the interferometer are spatially separated and independent of each other. This experimental setup has high flexibility for making modifications on each arm as it was done in previous works^26–29^. From a different point of view, however, the long and separated beam paths (about 2.9 m each) make precise control of the delay between the pump and probe pulses challenging: each optical element of the arms introduces vibrations and thermal drift, and therefore delay instabilities scale with the size and the amount of optics of the setup (see section “Attosecond interferometric stability”). The driving mid-IR beam is focused by a silver 90$^\circ$ off-axis parabolic mirror with a focal length of 250 mm into an HHG target. Such a parabolic mirror is suitable for tight focusing without introducing chromatic aberrations. The generated SXR beam is transmitted through a set of three pinholes to remove the driving mid-IR beam. The SXR is then transmitted through a thin metallic filter to remove the residual driving mid-IR beam and shape the SXR spectrum. The SXR beam is incident on a nickel-coated toroidal mirror at a grazing angle of 3$^\circ$ such that the generation focus is imaged in a 2*f*-to-2*f*-configuration, resulting in total separation between the two foci of 1.2 m. Thus, the SXR focus in the interaction chamber is estimated to be a 1:1 image of the focus in the generation region.

The pump beam is directed on a mirror pair fixed on top of a translation stage for varying the time delay during pump-probe measurements. After the delay line, the mid-IR beam is guided towards the rectangular recombination chamber, where it is focused with a parabolic mirror (*f* = 400 mm) onto the target sample, leading to a focal spot size of approximately 70 $\upmu$m. Since the pump beam is reflected from the perforated recombination mirror, $\sim$ 0.4 mJ reach the sample.

Pump-probe experiments strongly benefit from a shot-to-shot referencing method. By blocking one of the interferometric arms every millisecond and subtracting the reference spectrum, the noise originating from fast fluctuations could be significantly reduced. Unfortunately, up to now, it is hard to obtain enough photon flux from an SXR table-top source to perform shot-to-shot measurements. Thus, integration over multiple laser shots is used, and a mechanical shutter is implemented into the mid-IR-beam path to perform differential “pump on - pump off” measurements at each delay step. Reference spectra without sample gas are additionally recorded before and after each full scan.

Finally, the SXR and mid-IR beams are recombined at 90$^\circ$ with a perforated (two-millimeter hole) prism-like silver mirror and propagate co-linearly towards the sample target. Therefore, the length over which the pump and the probe beams propagate separately is about 2.9 m. Over such a distance, a small pointing fluctuation of each beam could lead to a spatial separation of the two foci. The spatial overlap of the two foci in the propagation direction is defined by the position of the HHG focus. The Rayleigh range of the SXR is long enough (237–713 mm for the experimentally observed photon energies) that if the HHG focus is moved by a couple of millimeters away from the optimal position, the focal positions of the pump and probe beams separate negligibly in the interaction region.

After the interaction region, the residual mid-IR beam is filtered by a 150-nm-thick aluminum filter (for most of the measurements), whereas the SXR beam is imaged by a flat-field concave grating on a vacuum charge-coupled device (CCD) camera (see section “High-resolution soft-X-ray spectrometer”).

### High-resolution soft-X-ray spectrometer

**Figure 2:**
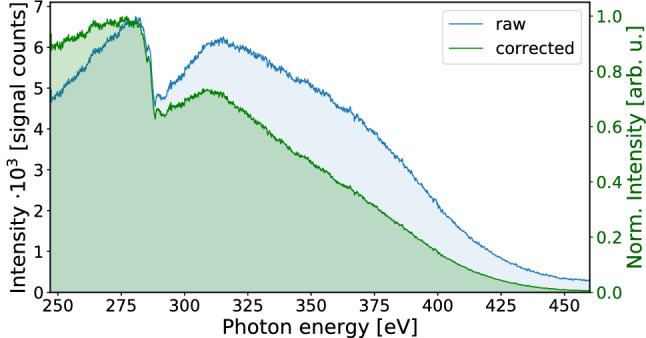
Typical measured raw (blue curve) and Jacobi-corrected (green curve) SXR spectrum generated in helium and transmitted through 150 nm Al filter. The integration time was set to 0.5 s and the camera gain to two. The absorption peak around 285 eV is due to carbon contamination of the SXR optics.

The SXR spectrum is dispersed by a flat-field grazing-incidence aberration-corrected concave grating (Hitachi, 001-0659) and imaged onto an X-ray back-illuminated CCD camera (Andor Newton, D0940P BEN). The grating rotation and its position can be adjusted to optimize the spectral resolution, while the camera can be moved along the grating focal plane to capture a certain part of the SXR spectrum. The ultimate resolution of the current spectrometer design is dictated by the SXR-source size along the dispersion direction (since the adjustable-width entrance slit is not used due to the associated intensity losses), by the grating groove density and its surface quality, by the distance of the CCD camera from the source and by the effective spatial resolution of the camera. The performance of the spectrometer was estimated from its detection efficiency and its spectral resolution. The resolution of our spectrometer has been determined by the FWHM of a gaussian fitting around the narrowest feature of the absorption profile in argon. Table 1 represents a summary for the spectral resolution obtained from the absorption profiles of argon, ethylene and carbonyl sulfide.

**Table 1:** Experimentally measured (this work) spectral resolution of the CCD-based spectrometer and (literature) values, extracted from literature. The resolving power ($R_p$) is given by: $R_P=\dfrac{Energy}{FWHM}$ and the resolution (*R*) is equal to $\dfrac{1}{R_p}$.

Atom/molecule	Edge	Energy (eV)	FWHM$_{lit}$ (eV)	FWHM$_{this~ work}$ (eV)	$R_{P,~lit}$	$R_{P,~this~work}$	$R_{lit}$ $10^{-3}$	$R_{this~work}$ $10^{-3}$
Ar	L$_{2,3}$ (2p$_{3/2}$)	244.3	0.150	0.164	1673	1490	0.600	0.67
C$_2$H$_4$	C K (1s)	284.8	1.000	1.13	1531	1490	0.653	0.67
OCS	C K (1s)	288.4	0.643	0.799	1531	1490	0.653	0.67

Thus, our home-built compact spectrometer using the variable-line-spacing concave grating and the vacuum CCD camera covers not only the energy range of the most abundant elements in organic molecules as carbon or nitrogen, but includes sulfur, chlorine, and argon, and it achieves a resolving power of around 1490 at the argon L$_{2,3}$-edges. Typical high-order harmonic spectrum obtained with the CCD-based spectrometer is shown in Fig. 2 (blue curve). The signal from the spectrometer is recorded per unit wavelength. To convert the wavelength axis to the energy axis, the intensity of the spectrum was scaled using the Jacobi transform ($\frac{d\lambda }{d~Energy}$, Fig. 2, green curve), to ensure the correct representation of the measured spectrum.

A direct measurement of the absolute value of the SXR photon flux from table-top light sources is not possible, therefore we estimate the photon flux in our setup based on a CCD-camera-based calibration (Fig. 2, green curve). The photon flux evaluation takes into account: grating efficiency (4.8% at 550^30^), Al filter transmission (taken from the Henke database^31^), camera integration time (0.5 s), camera quantum efficiency, camera sensitivity, camera quantum yield (3.63 eV for an energy range between 0.5 and 8 keV^32^), laser repetition rate. Under the aforementioned conditions, the estimated total SXR photon energy and the photon flux for the water-window region captured by the CCD camera chip are equal to [4.30 $\times \, 10^5$] ph/s at 280 eV for the 247.3–523.1 eV accessible energy range, respectively. The photon flux next to the carbon K-pre-edge is [1.53 $\times \, 10^4$]ph/s/1$\%$ bandwidth at 280 eV (range between 278.61 eV and 281.40 eV). Errors in the photon flux estimations could derive from the evaluation on the grating’s efficiency as a function of the photon energy, the filter transmission (a flat filter without oxidation contamination has been considered), and the carbon layer that could have been deposited on the camera chip. Johnson et al.^9^ reported that a thin film was deposited over time onto the face of their extreme-ultraviolet-sensitive camera. Therefore, one could expect a negative impact on the camera sensitivity and therefore an underestimated photon flux value.

### Attosecond interferometric stability

Attosecond temporal stability of the pump-probe setup is necessary for attosecond time-resolved experiments^27,29,33–38^. The time delay between the pump and probe pulses, traditionally introduced by splitting and recombining two pulses in an interferometer, suffers from instability in the relative path lengths. A change of the relative arm length by 30 nm already introduces a delay variation of 100 as this value is significant for experiments with a temporal resolution on the few-attosecond timescale. The instability of the pump-probe delay is mostly triggered by mechanical vibrations, thermal drifts of the optical elements, and fluctuating environmental conditions. The classical approach to achieve high stability is a passive stabilization that requires all mechanical components to be carefully attached to a single-piece temperature-stabilized optical table, and all optics be tightly fixed in their mounts. Possible vibrations coming from the turbomolecular pumps coupled to the vacuum chambers and roughing pumps located close to the experimental station are strongly attenuated by dampers.

**Figure 3:**
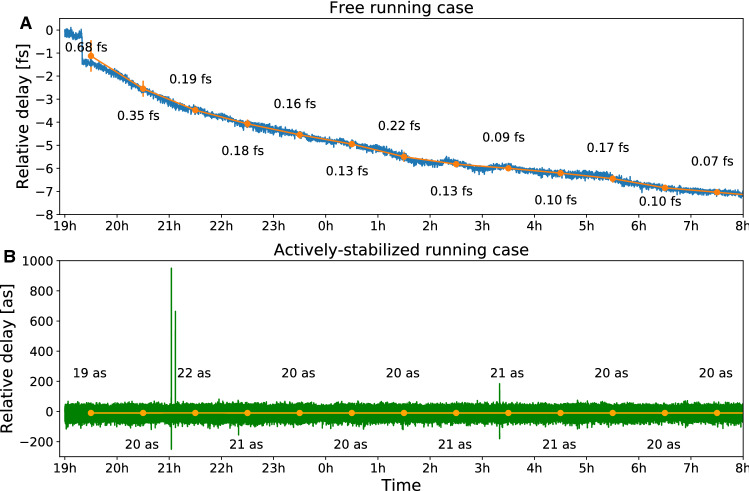
Relative delay in the SXR and mid-IR beam paths. Long-term stability of the relative delay in the SXR and mid-IR beam paths measured overnight for (**A**) the free-running case and (**B**) the actively-stabilized case. The reported numbers represent the RMS value of the measured relative phase over a period of 1 h (orange).

By coupling a continuous-wave (cw) HeNe laser beam into the Mach–Zehnder interferometer from the back side of the beam splitter and by co-propagating it through exactly the same pump and probe optics as the mid-IR and SXR beams, the interferometric stability is achieved through active stabilization. The interference fringes, resulting from the overlap of the cw beams propagating along the pump and probe arms are carrying information about the path-length difference between the two interferometric arms. In our case, the passive stabilization can provide stability of a few hundred attoseconds over an hour under the condition that human presence around the experiment (outliers between 19 and 20 o’clock shown in Fig. 3A) is suppressed and no other experiment is running on the same optical table. The long-term stability is limited to several femtoseconds over a period of a few hours (Fig. 3A). The free-running relative delays for short- and long-term measurements are obtained from in-loop measurements. Since there are no other optics after the pump-probe recombination, the free-running experimental relative delay is expected to be highly correlated to the delay measured by the HeNe reference interferometer.

Additionally, the cw-laser interferogram can be used to actively stabilize the relative delay between the SXR and mid-IR pulses on the attosecond time scale (below 50 as over several hours) as shown in Fig. 3B. In this case, most of the disturbances can be compensated, including shocks (outliers at around 21 and 3 o’clock shown in Fig. 3B) and long-term drifts. It is important to mention that the RMS value of the measured relative phase variation gives only the order of the interferometric stability and can be different on a day-to-day basis depending on the environmental conditions.

### Attosecond pulse duration

The pulse duration of the SXR probe pulse has not been directly measured during the present experiments. However, some of the present authors have used the same commercial OPA and a pulse compression system based on a hollow-core fiber and bulk material as reported in Gaumnitz et al.^6^, where they synthesized a 43-attosecond pulse. Cousin et al.^39^ have also synthesized isolated attosecond pulses with a sub-two-cycle, CEP-stable, 1.85 μm﻿, 1-kHz driving pulses for the carbon K-edge spectroscopy produced with similar equipment (OPA and hollow-core fiber compressor) as in our case. They reported a pulse duration of less than 322 as. Finally, Li et al.^40^ have reported the generation of 53-attosecond SXR pulses with 12 fs driving pulses centered at 1.8 μm﻿. Based on these results, we can safely agree with our estimation that our attosecond pulse duration is below 200 as.

### Divergence of high-order harmonics

**Figure 4:**
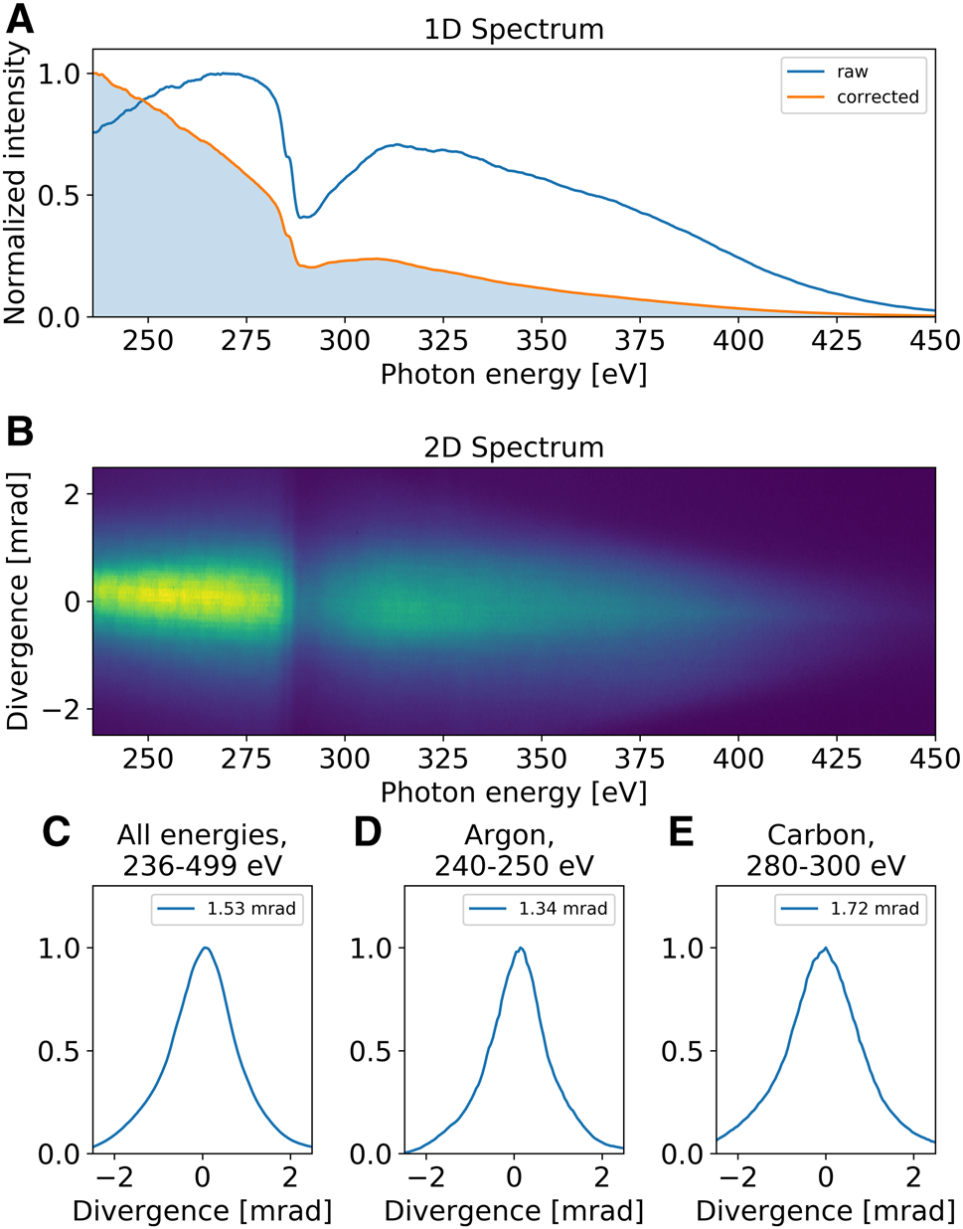
Spatially-resolved harmonic spectrum generated in helium. (**A**) Typical HHG spectrum with the photon flux optimized for measurements at the carbon K-edge. The blue curve is the raw data, the orange one has been corrected by the Jacobi factor and 150-nm-thick Al filter transmission. (**B**) 2D corrected-HHG spectrum, showing both the photon energy (horizontal axis) and divergence (vertical axis). Beam divergence profiles integrated over all energies detected by the CCD-camera chip (**C**); over 240–250 eV energy range at the argon L-edge (**D**); over 280–300 eV energy range at the carbon K-edge (**E**).

For the used focusing geometry, the harmonic flux is optimized by scanning the backing pressure of the generation target from 0 to 10 bar of helium. The cutoff energy is far beyond the carbon K-edge (285 eV), and the highest yield is reached together with the highest cutoff at a backing pressure of about 4 bar. The adjustable parameters for optimizing the SXR yield are the mid-IR beam diameter, the backing gas pressure, the mid-IR pulse energy, the mid-IR pulse compression with bulk material, the gas pressure in the hollow-core fiber, and the CEP. Figure 4A (blue curve) shows a typical energy-calibrated HHG spectrum obtained by tuning the aforementioned parameters such that the maximum photon flux is observed at 290 eV, suitable for revealing dynamics of carbon-based molecules^10^. The spectrum in Fig. 4A (orange curve) was corrected by the Jacobi factor, aluminum filter transmission, and normalized by the average overall energy values.

The generated SXR radiation is emitted with a small divergence compared to the driving mid-IR laser pulse, which follows from the short wavelength of the generated SXR light, together with the coherence between the emission process and the electric field of the driving laser pulse. The obtained spectrum is presented in Fig. 4B, which indicates a relatively small beam divergence across the entire spectral range. Importantly, the shown spectra are measured after re-focusing of the SXR beam by the toroidal mirror. The divergence values are obtained by taking into account the beam size of the SXR, the pixel size of the chip, the propagation distance, and the measured intensity profile. The grating efficiency as a function of energy is not included, but it has been corrected for the average overall energy as discussed above.

The divergence profiles for the detected spectrum are shown in Fig. 4C–E. For the energy range at the argon L-edge (Fig. 4D), the divergence is 1.34 mrad that is smaller than that of 1.72 mrad at the carbon K-edge (Fig. 4E). Averaged over all detected energies, the divergence is 1.53 mrad (Fig. 4C), which is comparable to other reported values^41^. The Gaussian-like profile indicates a good phase matching along the propagation direction of the driving mid-IR beam.

This work shows that by employing sub-two-cycle mid-IR laser pulses to drive HHG, it is possible to generate attosecond pulses that extend beyond 450 eV, covering almost the entire water-window energy region. Thus, the table-top HHG source developed in this work enables us to perform attosecond time-resolved X-ray absorption measurements in the energy range where the samples with biological or chemical relevance absorb. However, it is important to mention that the observation of the continuous SXR spectrum is not a complete proof of the production of isolated attosecond pulses. Temporal characterization of the generated SXR pulses is required but is beyond the scope of the current work.

## Spectral and temporal resolution of the instrument

In order to demonstrate the performance of our table-top SXR light source, static and time-resolved absorption measurements are discussed in this section.

### Measurement of static absorption spectra with the high-harmonic light source

**Figure 5:**
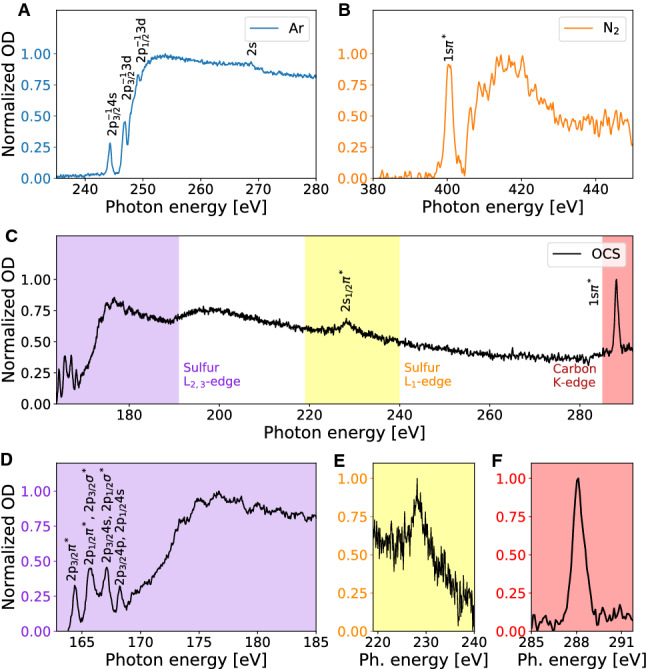
Static X-ray-absorption spectra of (**A**) argon at the L$_{1,2,3}$-edges, (**B**) diatomic nitrogen at the K-edge, and (**C**) carbonyl sulfide at the sulfur L-, and carbon K-edges simultaneously. Zoom of the carbonyl sulfide absorption spectrum at the sulfur L$_{2,3}$-edges (**D**), sulfur L$_{1}$-edge (**E**), carbon K-edge (**F**). The reported OD values have been normalized between zero and one per each region of interest.

The absorbance is defined as an optical density OD $=- \log _{10}\frac{I}{I_0}$, where *I* and $I_0$ are the background-corrected spectra measured with and without the sample, respectively. The corresponding absorption spectrum (OD) of argon at the L$_{1}$- and L$_{2,3}$ edges is shown in Fig. 5A. At the spectral resolution of 0.6 meV, three peaks in the series converging to the argon 2p$^{-1}$ threshold and one peak corresponding to argon 2s transition can be resolved^31,42–44^. Comparing our static absorption spectrum of argon with the spectra reported in^45–47^, we find them in good agreement and demonstrate the high-energy resolution of our apparatus.

Similarly, absorption spectra around nitrogen K-edge can also be measured (Fig. 5B). The broad spectrum of our SXR pulse allows us to experimentally observe several absorption edges simultaneously, a good example is carbonyl sulfide (OCS) whose static absorption spectrum is reported in Fig. 5C. Four absorption edges of two elements are within the spectral bandwidth of our attosecond pulse: sulfur L${_1}$-, L$_{2,3}$-, and the carbon K-edges (Fig. 5C). The spectroscopic assignment of absorption lines was performed from near edge X-ray absorption fine structure measurements^48,49^. The pink-shaded area in Fig. 5C (zoom in Fig. 5D) corresponds to the S L$_{2,3}$-edge with four strong resonance peaks “1”–“4” followed by a weaker Rydberg series (mostly convoluted spectrum due to the spectrometer resolution) converging to the ionization potentials (at 171 eV and 172 eV for L$_{3}$- and L$_{2}$-edges, respectively), and a shape-resonance structure (>177.5 eV) in the continuum above the ionization thresholds^49^. The first peak “1” in the total spectrum is solely excitation from the 2p$_{3/2}$(L$_{3}$) channel, while the peaks “2”-“4” are mixtures of excitations from both channels, 2p$_{1/2}$(L$_{2}$) and 2p$_{3/2}$(L$_{3}$). The yellow-shaded area in Fig. 5C (zoom in Fig. 5E) corresponds to the S L$_{1}$-edge (2s$_{1/2}$) with a single peak “5”. Its assignment is based on the fact that the L$_{1}$-edge of the atomic sulfur is at 229.2 eV^44^. However, the assignment of this transition was not found in the literature. The orange-shaded area in Fig. 5C (zoom in Fig. 5F) corresponds to the carbon K-edge with one prominent absorption peak assigned to the transition from the carbon core level to an unoccupied antibonding $\pi ^*$ orbital, C 1s $\longrightarrow \pi ^*$. Due to the design of the SXR spectrometer, we are able to observe higher energies at the carbon K-edge only at the expense of not catching the energies at the S L$_{2,3}$-edge. Starting from 291.4 eV, transitions to *n*s and *n*p ($n\ge$ 3) and at energies higher than 297.0 eV shake-up and $\sigma ^*$ shape resonances would be observed.

### Time-resolved measurement in argon demonstrating sub-cycle temporal resolution

**Table 2:** ATAS instruments used to measure dynamics in argon at L$_{2,3}$-edges.

	Chew et al. (2018)^45^	Barreau et al. (2020)^46^	This work
SXR Probe			
Driving central wavelength (nm)	1700	1300	1714
Driving pulse energy (mJ)	(1.8)	1.0	1.3
Driving pulse duration (fs)	< 11.4	12.8	< 11.0
Pump	Mid-IR	IR	Mid-IR
Central wavelength (nm)	1700	800	1714
Pulse energy (mJ)	(0.2)	0.8	0.4
Pulse duration (fs)	11.4	5	< 12.0

**Figure 6:**
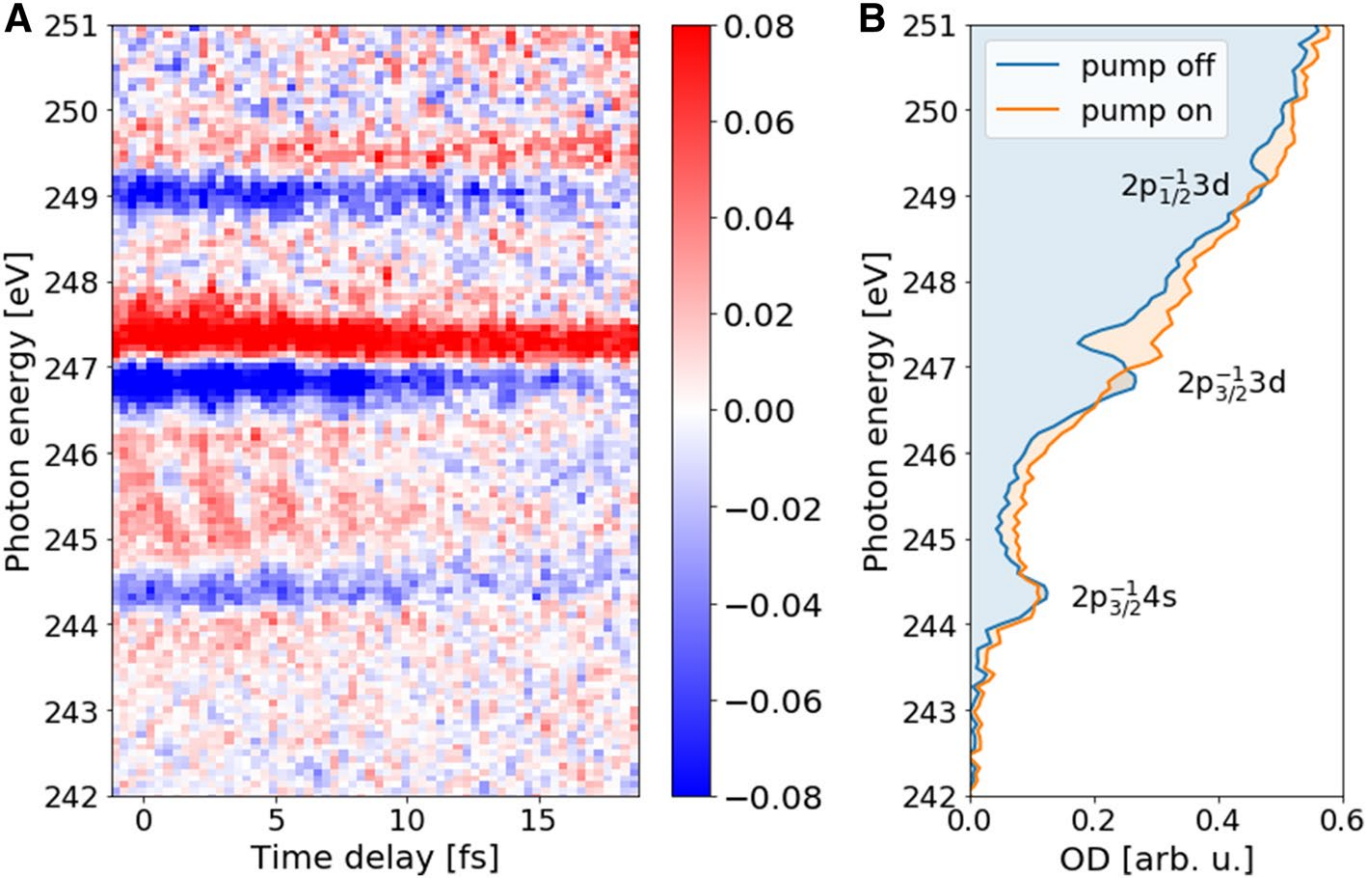
SXR absorption spectra of argon. SXR absorption spectra in the vicinity of L$_{2,3}$-edge of argon as a function of pump-probe delay (**A**). SXR-probe pulse precedes mid-IR-pump pulse. SXR absorption spectra without and with mid-IR pump (**B**).

Figure 6 shows the change in optical density ($\Delta$OD) in argon as a function of pump-probe time delay for the 2p$^{-1}_{3/2}$4s, 2p$^{-1}_{3/2}$3d, and 2p$^{-1}_{1/2}$3d states. At each delay point $\tau$, the change in optical density is calculated as $\Delta OD(\tau )=- \log _{10} \frac{I_{pump~on}(\tau )}{I_{pump~off}}$, where $I_{pump~on}(\tau )$ and $I_{pump~off}$ are the spectra collected with and without the delayed mid-IR beam, respectively. The pump intensity was adjusted to remain below the threshold to drive the HHG process in argon. The measurement was performed without the residual mid-IR beam from the high-harmonic generation. Negative delay values correspond to the SXR arriving first. The laser parameters of the present and previous experiments are summarized in Table 2. We note that Chew et al.^45^ in their experiment observed sub-cycle oscillations, whereas Barreau et al.^46^ did not observe them. In our experiment, we observe the sub-cycle oscillations as shown in Fig. 6. Moreover, with a step size of 333 as, we are capable of observing a spectral change in the signal from one delay step to the next. Therefore, we can estimate an upper limit of 400 as for the temporal jitter between the pump and probe arms of our transient-absorption beamline.

## Discussion

Up to date, there have been spectacular achievements in the development of tabletop SXR attosecond instruments^5,9,41^ allowing for performing time-resolved measurements at the carbon and nitrogen K-edges. In spite of this, the main factors limiting the realization of such experiments have remained the unstable, insufficient, and/or nonuniform flux of the SXR light source. However, with the present attosecond apparatus, we have obtained great temporal stability and a high signal-to-noise ratio, as was reported in Ref.^10^. The first attosecond time-resolved absorption measurements with this apparatus we performed in ethylene at the carbon K-edge. This work realized the simultaneous observation of the multidimensional structural dynamics and, most notably, the observation of the fastest electronic relaxation dynamics via a conical intersection. We have shown that the electronic relaxation from the first electronically excited state to the ground state of the ethylene cation takes place with a short time constant of $6.8\pm 0.2$ fs. We note that in a similar experiment, Saito et al.^50^ have shown great results in the real-time observation of electronic, vibrational, and rotational dynamics simultaneous in nitric oxide with their attosecond SXR apparatus at 400 eV. By employing SXR and two-cycle 1800 nm pulses, Smith et al.^47,51^ have observed prominent femtosecond dynamics at the carbon K-edge of liquid methanol and ethanol that was attributed to the valence-shell ionization dynamics.

We have demonstrated the attosecond transient-absorption spectroscopy instrument capable of delivering high spectral and temporal resolution, covering absorption edges of most light elements from silicon L-edge up to nitrogen K-edge, long-term stability, and high photon flux, which is a key for observing relatively small absorption changes induced by the pump pulse. We have shown that the ATAS technique is particularly powerful in revealing electronic dynamics at several absorption edges simultaneously. Moreover, at the same time, this technique can display the temporal evolution of the structural dynamics. These developments pave the way to the study of charge migration and charge transfer, as well as many other electronic processes in complex molecules, solvated molecules and advanced materials, adding attosecond temporal resolution to the powerful technique of soft-X-ray absorption spectroscopy.

## Data Availability

The datasets generated and analysed during the current study are available in Ref.^52^.
